# Genome-wide identification and characterization of *Solanum tuberosum BiP* genes reveal the role of the promoter architecture in BiP gene diversity

**DOI:** 10.1038/s41598-020-68407-2

**Published:** 2020-07-09

**Authors:** Venura Herath, Mathieu Gayral, Nirakar Adhikari, Rita Miller, Jeanmarie Verchot

**Affiliations:** 1 Texas A&M Agrilife Center in Dallas, Dallas, TX 77953 USA; 2 Department of Plant Pathology and Microbiology, Institute for Plant Genomics and Biotechnology, Texas A&M University, College Station, TX 77802 USA; 3 Department of Biochemistry and Molecular Biology, Oklahoma State University, Stillwater, OK 77845 USA; 4 Department of Agriculture Biology, Faculty of Agriculture, University of Peradeniya, Peradeniya, 20400 Sri Lanka

**Keywords:** Protein trafficking in plants, Abiotic, Biotic, Heat, Plant molecular biology, Chaperones, Endoplasmic reticulum

## Abstract

The endoplasmic reticulum (ER) immunoglobulin binding proteins (BiPs) are molecular chaperones involved in normal protein maturation and refolding malformed proteins through the unfolded protein response (UPR). Plant BiPs belong to a multi-gene family contributing to development, immunity, and responses to environmental stresses. This study identified three *BiP* homologs in the *Solanum tuberosum* (potato) genome using phylogenetic, amino acid sequence, 3-D protein modeling, and gene structure analysis. These analyses revealed that *StBiP1* and *StBiP2* grouped with *AtBiP2*, whereas *StBiP3* grouped with *AtBiP3*. While the protein sequences and folding structures are highly similar, these *StBiPs* are distinguishable by their expression patterns in different tissues and in response to environmental stressors such as treatment with heat, chemicals, or virus elicitors of UPR. Ab initio promoter analysis revealed that potato and Arabidopsis *BiP1* and *BiP2* promoters were highly enriched with cis-regulatory elements (CREs) linked to developmental processes, whereas *BiP3* promoters were enriched with stress related CREs. The frequency and linear distribution of these CREs produced two phylogenetic branches that further resolve the groups identified through gene phylogeny and exon/intron phase analysis. These data reveal that the CRE architecture of *BiP* promoters potentially define their spatio-temporal expression patterns under developmental and stress related cues.

## Introduction

One of the best characterized molecular chaperones in the endoplasmic reticulum (ER) is the ER binding immunoglobulin protein (BiP), also known as the glucose receptor protein 78 (GRP78), is conserved across evolutionary kingdoms. BiP guides the co-translational translocation of nascent proteins into the ER, and chaperones protein folding and maturation. BiP contains N-terminal nucleotide-binding domain (NBD) and C-terminal substrate-binding domain (SBD). The NBD has two lobes surrounding the allosteric ATP-binding site to modulate substrate binding. The SBD has SBDβ and SBDα subdomains to bind the hydrophobic surfaces of newly translated proteins to prevent aggregation. The SBDβ is a pocket with two primary loops that surround the nascent polypeptide and, the SBDα lid is covering this pocket^1^. All BiP proteins have a C-terminal HDEL or KDEL signaling motif for ER retention^2^.

Across eukaryotes, BiPs contribute to the unfolded protein response (UPR). Under normal condition, the mammalian BiP binds to and inhibits three ER stress sensors, protein kinase RNA-like ER kinase (PERK), activating transcription factor 6 (ATF6), and inositol-requiring enzyme 1α (IRE1α)^3,4^. BiP releases the sensors to activate ER-to-nucleus signaling cascades. BiP plays another key role in protein quality control by identifying and refolding misfolded proteins. The yeast Kar2p (BiP orthologue) binds to and inhibits the IRE1p, which is the master regulator of UPR. Dissociation of Kar2p/BiP releases the IRE1p/IRE1α to oligomerize and then splice the mRNA controlling production of the Hac1/XBP1 transcription factor^5^. In Arabidopsis, the ER stress sensors include IRE1a, IRE1b, bZIP28, and bZIP17. The IRE1a and IRE1b splice the mRNA controlling production of the bZIP60 (Hac1p/XBP1 orthologue) transcription factors. The bZIP28 and bZIP17 are transcription factors with transmembrane domains that are cleaved at the ER upon activation. In Arabidopsis, BiP regulates cleavage and removal of the bZIP28 transmembrane domain for nuclear translocation of the transcription factor^6,7^.

In contrast to yeast and mammals, plant *BiP* genes belong to a multi-gene family. The Arabidopsis, wheat (*Triticum aestivum*), citrus (*Citrus sinensis*), soybean (*Glycine max*), pepper (*Capsicum annuum* L.) and tobacco (*Nicotiana tabacum*) encode three or four BiPs which have been annotated and shown to help protect against many abiotic stresses including heat, cold, salt, heavy metal and osmotic stresses^17–21^. These BiPs also play crucial roles in biotic stress resistance and plant innate immunity^1–5,16^. Several studies link BiPs to the regulation of ER stress-mediated cell death^6–9^, hypersensitive cell death, and non-host HR programmed cell death induced by *Xanthomonas oryzae* pv. *Oryzae* in rice*, Pseudomonas syringae* pv. *maculicola* in Arabidopsis, and *Pseudomonas syringae* pv. tomato in soybean and tobacco, *Phytophthora sojae* in soybean^10–14^.

*Arabidopsis thaliana* has three well-studied *BiP* proteins. AtBiP1 and AtBiP2 are almost identical (99% protein identity) and ubiquitously expressed^8,9^. AtBiP3 is less conserved (80% protein identity with AtBiP1/2) and is only expressed in response to ER stress^6,8^. *AtBiP1* and *AtBiP2* expression correlates with the synthesis of seed storage proteins and is induced in flower organs and endosperm tissue^9,10^. These AtBiPs also mediate fusion of polar nuclei during male and female gametogenesis^11–13^. The three Arabidopsis *BiP* promoters have *cis*-regulatory elements (CREs) known as the unfolded protein response element (UPRE) and ER-stress response element (ERSE) that are transcription targets of bZIP60, bZIP28 and bZIP17^8,14^. The *AtBiP* promoters have many other CREs for multiple factors having an additive effects on the levels or timing of gene induction^15^.

Although the *Solanaceae* family includes several agriculturally important crops, the *BiP* gene family in *Solanaceae* species remains largely unexplored, poorly annotated, and only partially characterized. This leaves a large gap in our knowledge about one of its major stress response pathways, the UPR. The first reported partial or complete cDNAs for tobacco BiPs identifying a multigene family was in 1991^15^. This report was prior to complete genome sequencing an annotation of any *Solanaceae* species. Six identified genes were classified based on the uniqueness of their 3′ untranslated sequences and were named BiP-like protein (BLP)1, BLP2, BLP3, BLP4, BLP5, and BLP8. Certain BLP proteins were reported to show tissue-specific patterns of expression, stress-related expression, and responsiveness to ER stress induced by tunicamycin treatment^2,15^. Newer studies reported the cDNA sequences for three *BiP* homologs in *C. annuum* and *S. lycopersicum* and the annotated loci are publicly available in the genome databases. Recent studies reported the cDNA sequences for four BiP homologs in *N. benthamiana* and *N. tabacum* and named them based on homology with *S. lycopersicum* or *Arabidopsis* BiPs^12,16^. While the transcripts were identified, the loci sequences for these *Nicotiana spp*. are incomplete. Furthermore, the putative transcript or loci IDs provided in databases have significantly changed since the first reported tobacco BiPs making it difficult to cross-reference recently reported sequences with historic information.

The potato genome sequence was first published in 2011 and the genome sequence consortium has been actively updating the sequence accessions (solgenomics.net). Potato is the third most important global food crop in terms of human consumption (CIP; https://www.cipotato.org) and is threatened by many fungal, bacterial, and viral pathogens for which resistance likely requires activities of the BIP gene family. This study identifies and characterizes the *BiP* gene family in potato (*Solanum tuberosum*), that we now know encompasses three BiPs. We analyzed their evolution, structural features, promoter architecture, and their expression under both development and stress-induced conditions. We report new insights that suggest their promoter architecture is key to differentiate the various roles of potato *BiP*s in development and during ER stress.

## Results

### Identification and phylogeny of StBiP

Arabidopsis BiP proteins (AtBiP1-3) were used to query Ensemble Plants and Solanum Genome Network (SGN) and to find homologs in *Solanaceae* (*Capsicum annuum*, *Nicotiana attenuata, N. benthamiana, N. tabacum, Solanum lycopersicum, S. tuberosum*)^17,18^. We also retrieved a broader set of putative plant BiP homologs for *Brachypodium distachyon, Glycine max, Oryza sativa*, *Sorghum bicolour, Triticum aestivum, Zea mays* from Ensemble Plants. Proteins were selected that satisfied the e-value of e^−25^. Candidate proteins were categorized as functional BiPs based on three predefining criteria: (1) predicted localization to the ER; (2) five internal domains for ATP hydrolysis and substrate binding; (3) ER retention signal (XDEL). Forty-six BiPs were recovered from both searches. Most BiPs were in the range of 639 to 678 amino acids and their molecular weights were between 71 and 75 kDa. There was one soybean BiP that was 581 amino acids in length and 63.7 kDa and another *N. benthamiana* BiP that was 851 amino acids and 95.8 kDa. The protein PIs were generally between 4.71 and 5.03 (Table 1). We identified three putative *S. tuberosum* BiPs as PGSC0003DMG400012254, PGSC0003DMG400018544, and PGSC0003DMG400024707 (Table 1 and Supplementary Table S1 online). These were renamed *StBiP1*, *StBiP2*, and *StBiP3* respectively. The amino acid sequence of StBiP1 and StBiP2 are highly similar (92%), whereas StBiP3 is more distantly related to StBiP1 and StBiP2 (75%).

**Table 1:** Molecular characterization of *Arabidopsis thaliana* and *Solanaceae* genes.

Species	Locus ID	Transcript ID	Gene name	MW (kDa)	PI	Length
*A. thaliana*	AT5G28540	At5G28540.1	*BiP1*	73.63	4.81	669
	AT5G42020	At5G42020.1	*BiP2*	73.56	4.84	668
	AT1G09080	AT1G09080.1	*BiP3*	75.15	4.68	675
*S. tuberosum*^a^	PGSC0003DMG400012254	PGSC0003DMT400031937	*StBiP1*	73.46	4.74	667
	PGSC0003DMG400018544	PGSC0003DMT400047710	*StBiP2*	73.61	4.80	668
	PGSC0003DMG400024707	PGSC0003DMT400063543	*StBiP3*	74.68	5.03	669
*S. lycopersicum*^b^	Solyc08g082820.3	Solyc08g082820.3.1	*SlBiP1*	74.78	5.03	670
	Solyc03g082920.3	Solyc03g082920.3.1	*SlBiP2*	73.49	4.77	668
	Solyc01g099660.3	Solyc01g099660.3.1	*SlBiP3*	73.91	4.77	673
*N. tabacum*^b^	Nitab4.5_0003658g0020.1	Nitab4.5_0003658g0020.1		74.5	4.84	667
	Nitab4.5_0004891g0060.1	Nitab4.5_0004891g0060.1	*NtBiP3a*	74.48	4.88	668
	Nitab4.5_0005771g0020.1	Nitab4.5_0005771g0020.1	*NtBiP3b*	73.8	4.79	668
	Nitab4.5_0009965g0010.1	Nitab4.5_0009965g0010.1		74.64	4.74	678
*N. benthamiana*^b^	Niben101Scf03115g02008.1	Niben101Scf03115g02008.1	BiP4	71	4.94	639
	Niben101Scf02755g06016.1	Niben101Scf02755g06016.1		72.1	4.78	655
	Niben101Scf02972g05008.1	Niben101Scf02972g05008.1		73.54	4.78	666
	Niben101Scf00369g07016.1	Niben101Scf00369g07016.1	BiP3b	95.77	4.71	851
*N. attenuate*	A4A49_57922	OIT38496	BIP5_0-1	73.35	4.81	666
	A4A49_23212	OIS99563	BIP-1	73.45	4.82	666
	A4A49_38137	OIT04096	MED37A-1	74.48	4.85	667
	A4A49_65725	OIT22272	BIP5_1	73.74	4.78	668
*C. annum*	T459_34102	PHT62035	BiP3	74.33	5.01	666
	T459_09640	PHT87534	BiP2	73.54	4.75	668
	T459_00063	PHT92181	BiP1	73.45	4.84	666

^a^StBiP1 and StBiP2 share 92% of amino acid identity with each other and 75% identity with StBIP3.

^b^The amino acid and nucleotide sequences for BiP homologs in *N. benthamiana*, *N. tabacum*, and *S. lycopersicum* were reported in Liebrand et al. and Jing et al. however the gene accessions were not reported^33,37^. For *S. lycopersicum*, we identified the same sequences in the SolGen database and we report the Locus and Transcript IDs. However, the *N. benthamiana* and *N. tabacum* BiP sequences reported in these prior studies had significant gaps and we could not attach common names to the gene sequences that are available in the current genome databases by cross-referencing to this earlier work.

^c^The *C. annuum* BiP genes are annotated here from Ensemble Plant and were reported in Wang et al., using their Sol Genomics Locus ID: CaBiP1(CaHsp70-8, CA01g00570), CaBiP2 (CaHsp70-7, CA03g20120), and CaBiP3 (CaHsp70-10, Capana08g001522)^17^.

**Figure 1:**
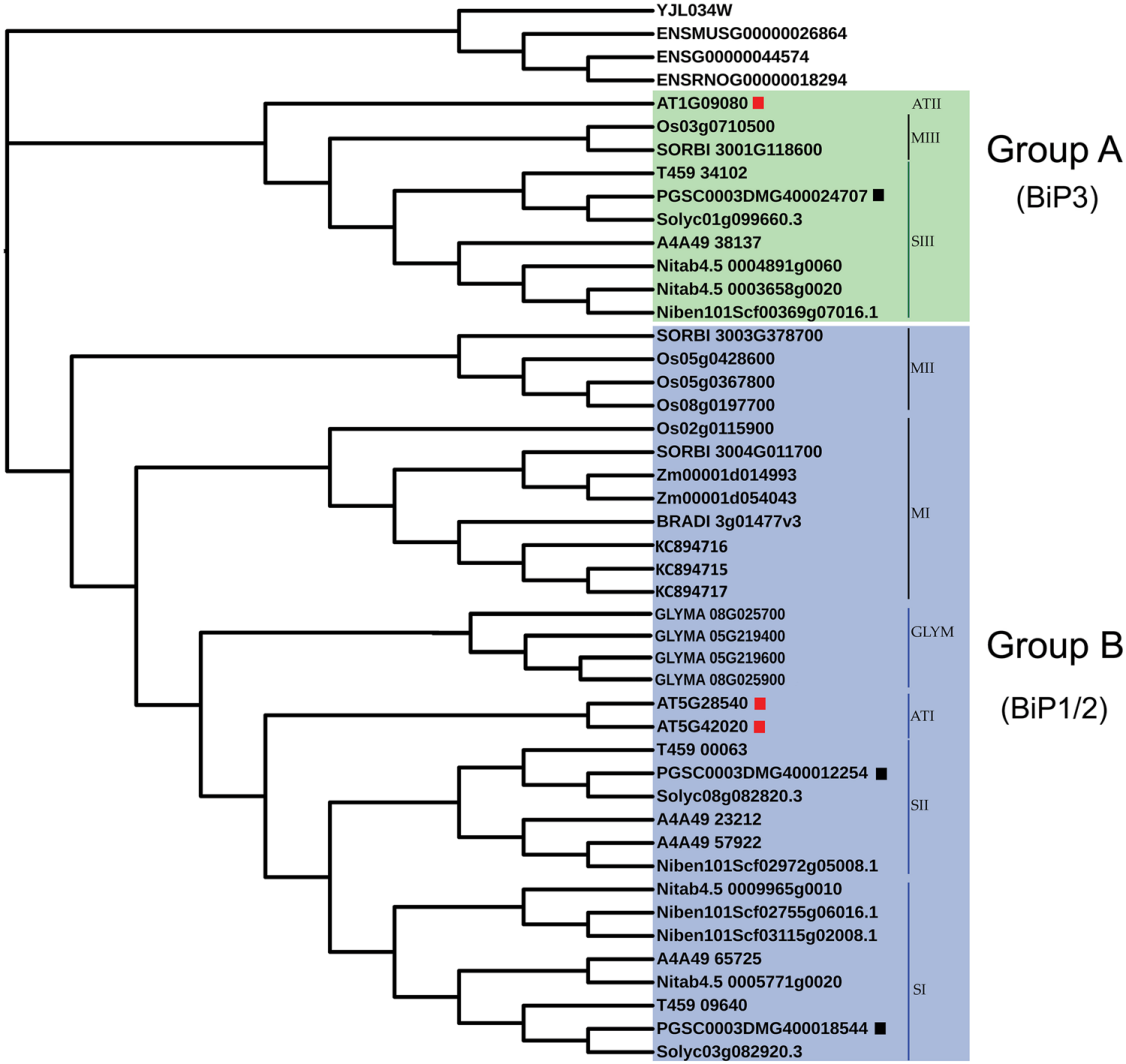
Phylogenetic tree constructed using the PhyML method (v1.5) with Seaview (v4.7) (https://doua.prabi.fr/software/seaview) contains BiP proteins among fungi, plants, and animals. The output was visualized using iTOL (v4) and Adobe Photoshop CC (2017). Group A is highlighted in green and contains BiPs that cluster with the AtBiP3. Group B is highlighted in blue and represents a larger group. The identified subgroups include three monocot (MI, MII, and MIII), two *Solanaceae* (SI and SII) and soybean and Arabidopsis (GLYM and ATI). The *AtBiP*s and *StBiP*s are marked with red or black squares, respectively.

We constructed a phylogenetic tree using PhyML (v. 1.5) with 1,000 bootstraps (Fig. 1) which included the 41 plant *BiP*s as well as five vertebrate and *Saccharomyces cerevisiae BiP*s (Table 1 and Supplementary Table S1 online)^19^. The phylogenetic tree included three branches representing plants, animals, and fungi. The plant *BiP*s were aggregated in two groups. Group A has ten *BiP*s belonging to monocots and dicots and, includes the *AtBiP3* and *StBiP3*. Surprisingly, there were no *B. distachyon, Z. mays, or G. max* genes represented in Group A. The monocot and *Solanaceae BiP3*-like proteins clustered in two sub-groups (MIII and SIII). These results indicate that there is an *AtBiP3* specific lineage represented in *Solanaceae*.

Group B was more diverse (Fig. 1) and the monocot and *Solanaceae* BiPs each clustered into two separate subgroups (MI, MII, SI and SII). The Arabidopsis and soybean BiPs clustered into distinct sub-groups (AT1 and GLYM). StBiP1 and StBiP2 were present in two *Solanaceae* sub-groups (SI and SII) closely related to the AT1 sub-group.

### Intron/exon structure of *BiP* genes

**Figure 2:**
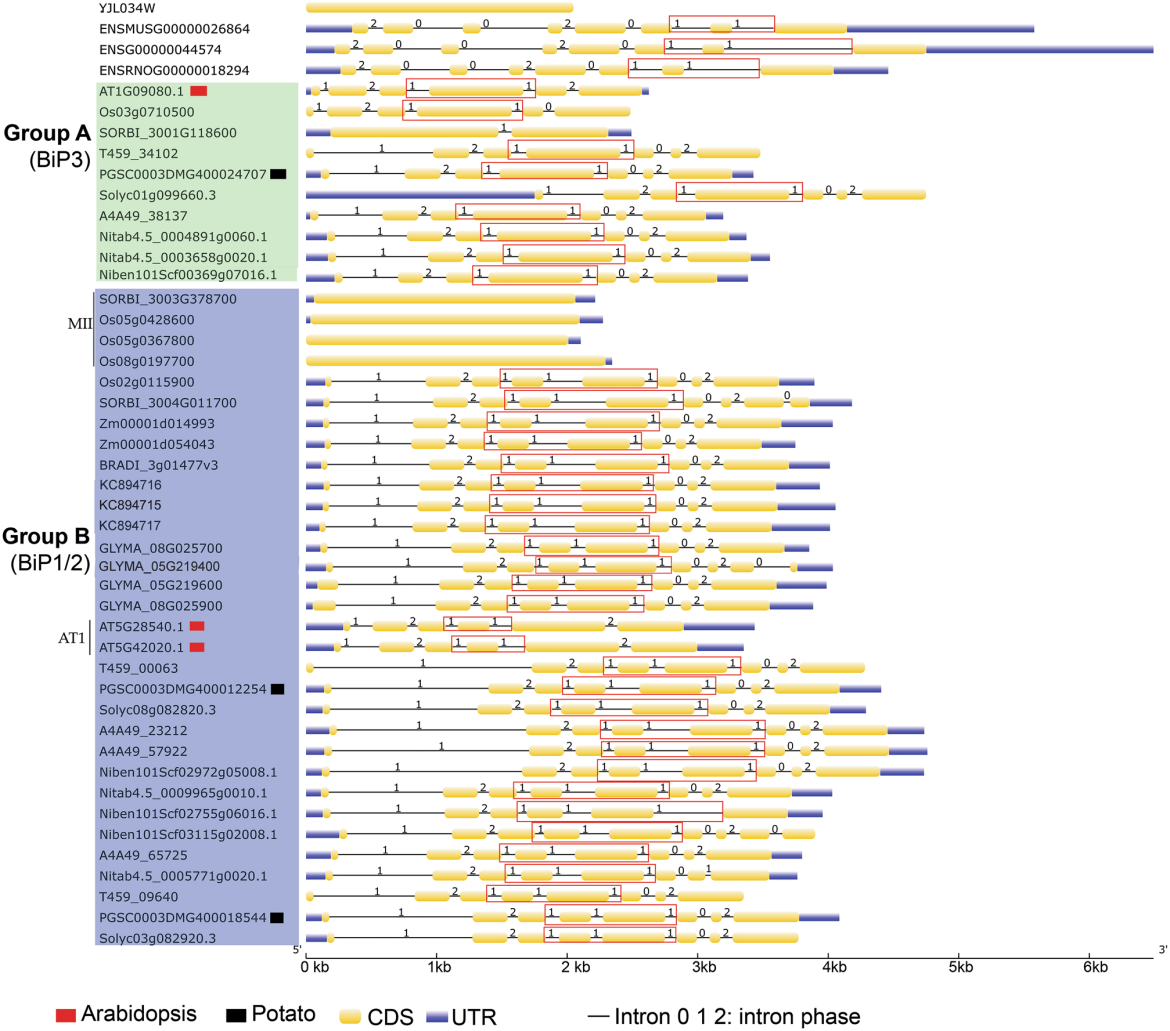
The structures of *BiP* genes, generated using GSDS 2.0 (https://gsds.cbi.pku.edu.cn/), starting from the transcription start site to the end of 3′ untranslated region in fungi, plants, and animals. Group A and Group B BiPs identified in green and blue boxes. The *AtBiP*s and *StBiP*s are marked with red or black squares, respectively, as in Fig. 1. The linear representation of each gene includes blue bars indicating 5′ and 3′ untranslated regions, yellow regions representing exons, and lines representing introns. The intron phase 0, 1, 2 are identified above each line. Red boxes surround the (1,1) symmetric exons. Most genes have one or two symmetric exons.

The intron positions and the frequency of intron phase combinations in related genes provide evidence of a common progenitor. Introns acquired in a progenitor and stabilized through evolution, maintain a non-random pattern. A random phase distribution of introns suggests that exons were shuffled through evolution, possibly creating new elements within gene products. The position of introns within a codon phase 0, 1, or 2, were mapped for the 46 *BiP*s (Fig. 2). The gene sizes of the 46 *BiP*s vary from 2,049 bps to 6,491 bps (Fig. 2). The yeast *BiP* (YJL034W) lacks introns. The representative animal *BiP*s possess seven introns with similar phase patterns suggesting a common origin. Notably, animal *BiPs* have intron phase 2 at the first intron while the majority of plant BIPs have phase 1 as the first intron. Near the 3′ end is a (1,1) symmetric exon. Within Group A genes, the *SbBiP3* (SORBI_3001G118600) stands out because it contains one intron and two exons. All other Group A genes have a central (1,1) symmetric exon surrounded by asymmetric exons in (1,2), (2,1), (1,0) and (0,2) class. The *AtBiP3* (AT1G09080.1) has a (1,2) asymmetric exon and both *AtBiP3* and *OsBiP3* (Os03g0710500) lacked the (0,2) asymmetric exon. The *AtBiP3* had a (1,2) asymmetric exon not found in other Group A genes.

Group B includes an assortment of genes contain between five and eight exons and the MII subgroup of the intron-less rice and sorghum genes (Fig. 2). The AT1 subgroup contains the *AtBiP1* and *AtBiP2* genes which have the least number of exons (5) primarily comprised of (1,2), (2,1) (1,2) asymmetric exons and a single (1,1) symmetric exon. Most Group B genes have two (1,1,) symmetric exons in the central region, a (1,2) symmetric exon near the 5′ end, and (0,2) or (0,1) asymmetric exon near the 3′ end (Fig. 2). The common intron patterns and phases suggests a common ancestral origin for most of the Group A and Group B BiPs.

### StBiP and AtBiP domain structures and amino acid sequences are highly conserved.

**Figure 3:**
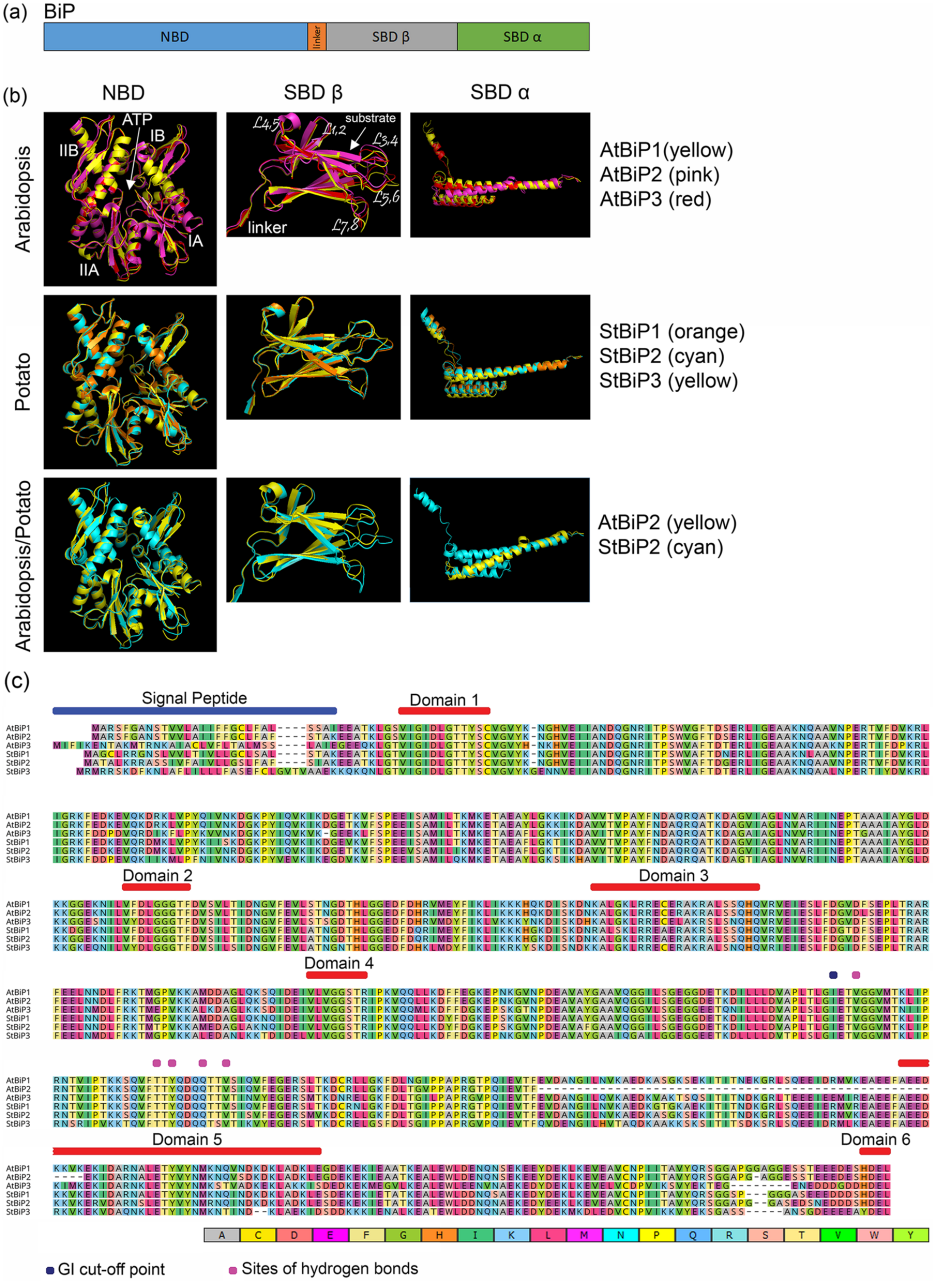
Domain structure of *Arabidopsis* and potato BiPs. (**a**) Schematic structures of the BiP domains. (**b**) The 3-D models are superimpositions of NBD, SBDα and SBDβ domains of AtBiPs and StBiPs and were generated using PyMol (v1.7.4) (https://pymol.org/). (**c**) Multiple sequence analysis of the AtBIP and StBiP proteins. The signal peptide, Domain1(β motif), Domain2 (γ motif), Domain3 (calmodulin-binding site), Domain4 (adenosine-binding motif), Domain5 (αβ motif), Domain6 (ER retention signal) are identified by bars above the alignment. The identical amino acids are identified by colors pointing to the high conservation among these proteins.

In all BiPs, the N-terminal nucleotide-binding domain (NBD) and C-terminal substrate-binding domain (SBD) are connected by a linker sequence that controls allosteric interactions^20,21^. The SBD has two subdomains SBDβ and SBDα (Fig. 3a). To compare the new plant BiPs with canonical structures, we used the I-TASSER server^22^ to generate three-dimensional structures of AtBiPs and StBiPs using the human and Chinese hamster BiPs as the threading templates (Fig. 3b; Supplementary Table S2 online). Supplementary Table S2 (see online) provides the confidence score (C-score), the TM-score and the RMSD values to explain the quality of the models for yeast, Arabidopsis and potato BiPs. The TM score and RMSD value correlate with the C-score^22,23^.

Next, we performed 3-D model superimposition of the well-characterized AtBiPs and candidate StBiPs (Fig. 3b) by overlaying the NBD, SBDα, and SBDβ to identify similarities. There are four structural subdomains oriented around one or more β-sheets (IA, IB, IIA, IIB) that divide the NBD into two lobes surrounding a central ATP binding pocket. This general folding structure is highly conserved among the AtBiPs and StBiPs with only minor variation (Fig. 3b). The SBDβ consists of eight β-strands forming a two-layered sandwich with five upper β-sheets and the three lower β-sheets. The SBDβ structure and amino acid sequences of the three AtBiPs and StBiPs are conserved. The SBDα consists of four helices. The 3-D models show that AtBiP2 has a shorter α-helical domain than AtBiP1 and AtBiP3. This corresponds to the gaps in the amino acid alignments that exist upstream of the domain 5 sequence (Fig. 3b, c). For all AtBiPs and StBiPs, except AtBiP2, the SBDα structure is conserved.

The I-Tasser predicted structures are consistent with amino acid conservation in sequence alignments of the candidate StBiP1, StBiP2 and StBiP3 proteins and other well-annotated plant BiPs. The first sequence alignment includes only Arabidopsis and *S. tuberosum* BiPs (Fig. 3c). The second alignment includes 46 BiPs used in the phylogenetic study (Supplementary Fig. S1 online). These alignments show the key conserved amino acid motifs that characterize members of the BiP gene family (Fig. 3c and Supplementary Fig. S1 online). Within the NBD and SBD are six amino acid sequence motifs that are remarkably well conserved among the 46 BiP sequences and are present in the StBiPs. The NBD contains the highly conserved β (Domain 1), γ (Domain 2), and adenosine binding (Domain 4) which provide the necessary ATPase binding and hydrolysis functions. The putative calmodulin-binding motif (Domain 3) located in the ATPase domain is also conserved among the StBiPs. The SBD contains the αβ domain (Domain 5), which includes a five- residues substrate-binding core that facilitates hydrogen-bonding with the peptide-substrate backbones. Finally, the StBiP1 and StBiP2 contain an ER retention HDEL signal sequence and StBiP3 contains a YDEL signal sequence.

### Potato *BiP*s are differentially expressed under developmental and stress conditions

**Figure 4:**
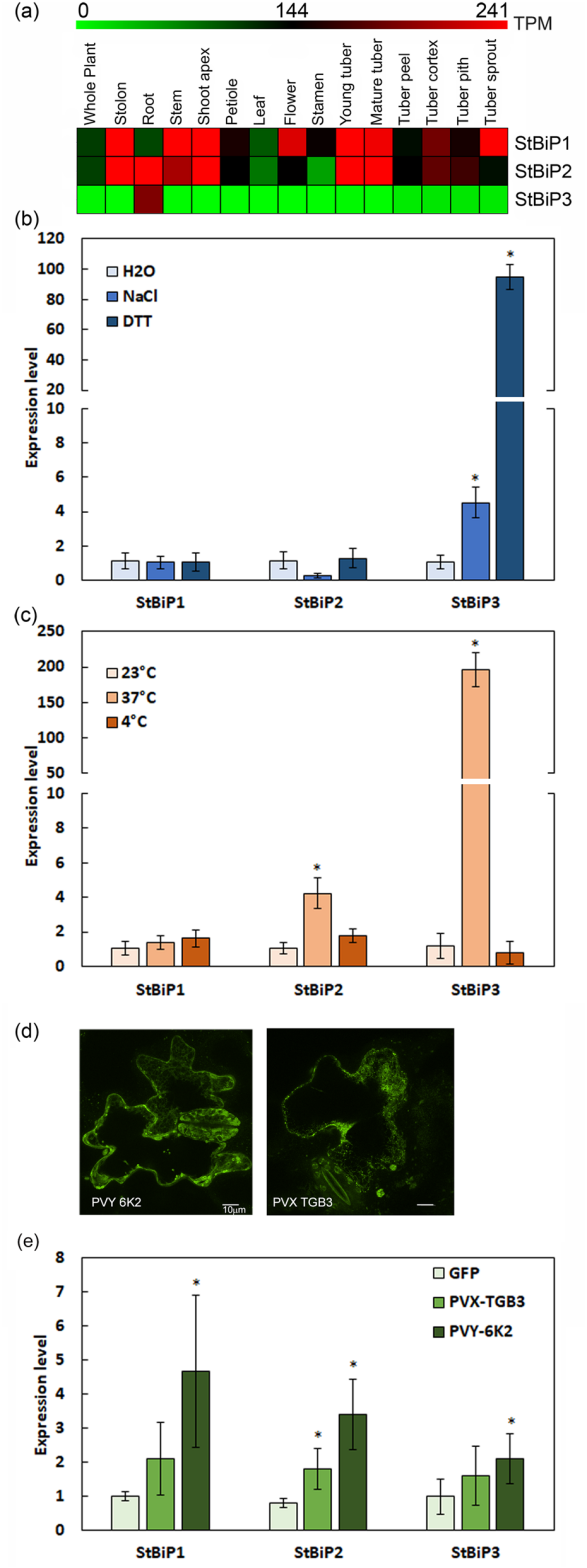
Expression profiles of *StBiP*s under developmental and stress conditions. (**a**) A heat map of tissue-specific expression profiles generated from RNA-seq data using MeV (v4.9.0) based on ArrayExpress accession E-MTAB-552^24^ (https://mev.tm4.org/). The transcripts per million (TPM) values of representative transcripts were used to generate the heatmap. The color scale above heatmap shows the expression levels; red indicates high transcript abundance while green indicates low abundance. (**b**–**d**) The bar graphs show the average *StBiP*s transcript levels determined by RT-qPCR following: (**b**) treatment with 2 mM DTT and 150 mM NaCl; (**c**) heat (37 °C) and cold (4 °C) treatment; (**d**) confocal images showing transient expression of GFP fusions with PVY 6K2 and PVX TGB3. (**e**) transient expression of the potyvirus 6K2 and the potexvirus TGB3 proteins. *Significantly different from the control (H_2_O, 23 °C and GFP); student t test; *p* < 0.05; n = 6).

We analyzed the three *StBiP* expression profiles using the publicly available RNA-seq data that was generated from 15 different organs and tissues and presented the data as heatmaps (Fig. 4a)^24^. Under normal conditions, *StBiP1* and *StBiP2* are constitutively expressed in all tissues and generally show the same pattern of moderate to high expression in stolon, stem, shoot apex, petiole, young and mature tubers. *StBiP1* is highly expressed in flowers while *StBiP2 and StBiP3* are highly expressed in roots under normal growth and development.

Since the UPR is an early and rapid response to environmental stress, we investigated whether these genes are upregulated to mitigate abiotic and biotic stress. Extensive prior studies of UPR related gene expression in *Arabidopsis thaliana*, *Glycine max, Nicotiana* species, and *Solanum* species treated leaves with DTT or NaCl. Time course experiments showed that the transcriptional response of several UPR related genes including *BiP* can be measured by RT-qPCR between 1 and 4 h after treatment^25–30^. We infiltrated potato leaves with 2 mM DTT or 150 mM NaCl to induce ER stress^30,31^. After four hours, the transcript levels of *StBiP1* and *StBiP2* remained unchanged while the *StBiP3* transcripts increased 4.5-fold following salt treatment and 95-fold following DTT treatment above the mock control (H_2_O; Fig. 4b).

Prolonged heat and cold treatment are commonly used to measure gene responses to persistent ER stress^28^. Gene expression during persistent ER stress influences plant growth and pro-survival activities. BiP is typically considered to be a core component of the UPR during both early and prolonged responses to ER stress^32^. Here we exposed potato plants to cold (4 °C) for 16 h and gene expression was unaltered (Fig. 4c). Potato plants were exposed to heat (37 °C) stress for 16 h^1^ and there was a fourfold increase in *StBiP2* and an approximately 200-fold increase in *StBiP3* in the leaves (Fig. 4c). Biotic stress was applied by delivering viral proteins that are well known to be specific inducers of the UPR^3,27^. Binary vectors expressing the potato virus X (PVX) TGB3, potato virus Y (PVY) 6K2 proteins fused to GFP, or GFP alone (negative control) were delivered by agro-infiltration to potato leaves. We confirmed fluorescent protein expression using confocal microscopy (Fig. 4d). At 4 days, leaves expressing the PVY-6K2 displayed elevated levels of *StBiP1*, *StBiP2*, and *StBiP3* transcripts that were between 2.1- and 4.7-fold higher than the GFP controls (Fig. 4e). Among the leaves expressing the PVX-TGB3, only *StBiP2* was significantly induced. These combined data indicate that the expression of *StBiP1* and *StBiP2* are responsive to heat or viral factors while the expression of *StBiP3* is responsive to DTT, NaCl, heat and viral factors.

### *StBiP* promoters are enriched with developmental, hormone-response, and stress-related transcription factor binding sites

To identify the basis of differential gene expression, we selected 1,000 bp upstream of the predicted transcription start site for the *StBiP* and *AtBiP* promoters and derived the predicted cis-regulatory elements (CREs). CREs belonging to 30 families of transcription factors were identified. Across all the Arabidopsis and potato *BiP* promoters, there are between 22 and 24 combined basic leucine zipper (bZIP), heat shock transcription factor (HSF), myeloblastosis (MYB) and flowering time regulator SQUAMOSA-PROMOTER BINDING PROTEIN-LIKE (SPL) transcription factor binding sites. Notably, the *StBiP1* lacks an HSF binding site, the *StBiP3* lacks the SPL binding site.

**Figure 5:**
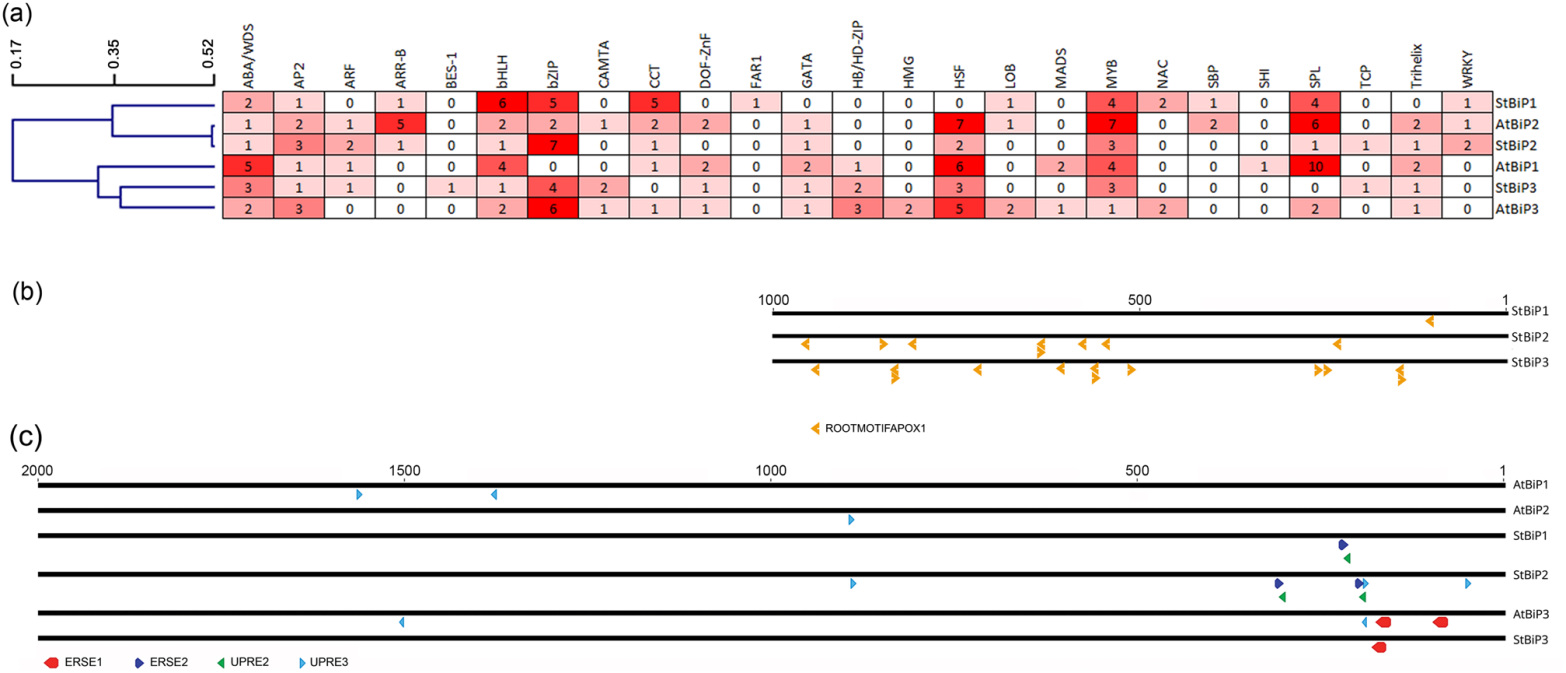
Distribution and frequency of CRE and ER-stress responsive elements on *AtBiP* and *StBiP* promoters. (**a**) The hierarchical clustering of CREs belongs to various transcription factor families identified using the TRANSFAC database release 2019.2. The average linkage distance was determined by hierarchical clustering and is presented on the right. The grid provides the number of sites that contain the CREs representing 25 transcription factor families. Shades of red were used to point to CREs that were highly represented (dark red), moderately represented (medium red), were represented once or twice (pale red), or not represented (white). The names of the promoters are identified on the right. (**b**) The distribution of ROOTMOTIFTAPOX1 (ATATT) elements (red arrows) in the 1,000 bp StBiP promoters are critical for root-preferential expression. These were promoters identified using the New PLACE database (https://www.dna.affrc.go.jp/PLACE). The promoter names are provided on the left. (**c**) The distribution of ER-stress responsive UPRE and ERSE in the 2000 bp AtBiP and StBiP promoters. The lines are drawn to scale with the nucleotide position relative to the transcription start site, represented above the lines. The colored arrows represent the positions of the CREs that are engaged by factors in the unfolded protein response. The names of these CRES are identified in the legend. The promoters are named on the right.

Hierarchical clustering of *StBiP*s and *AtBiP*s identified two promoter groups based on the presence and the distribution of CRE in their promoters (Fig. 5a). One cluster consisted of the *StBiP1*, *StBiP2, and AtBiP2* promoters. The second cluster consisted of the *AtBiP1*, *AtBiP3 and StBiP3* promoters (Fig. 5a). The architectures of the *AtBiP2* and *StBiP2, and* of the *StBiP3* and *AtBiP3* promoters were surprisingly conserved.

We identified key transcription factor families and CREs for differential gene expression during cell differentiation, organ development, and hormonal responses required for development (Fig. 5a–c and Supplementary Table S3 online)^1,2^. Regarding the MYB transcription factor family, the *StBiPs* and *AtBiP1* and *AtBiP2* the analysis identified: (a) MYB3R CREs that are components of the DP, Rb-like, E2F, and MuvB (DREAM) complex; (b) MYB24 CREs in *StBiP1*, *AtBiP1*, and *AtBiP2* promoters for gibberellic acid and jasmonic acid-mediated stamen development; and (c) WEREWOLF CREs in the AtBiP2 promoter for epidermal cell patterning. The root specific ROOTMOTIFTAPOX1 motif is distributed among all three *StBIPs* with 13 copies in the *StBiP3*, eight copies in the *StBiP2* and only one in the *StBiP1* promoters (Fig. 5b). The copy number of root specific CREs correlates with the respective high, medium, and low levels of *StBiP3, StBiP2* and *StBiP1* in roots seen in Fig. 4a.

Several large families of transcription factors have known roles in key developmental and metabolic processes in response to adverse environments including temperature and water stress. These include the abscisic acid stress and ripening/water deficit stress (ABA/WDS), APETALA2/Ethylene-responsive factor (AP2/ERF), and basic helix-loop-helix (bHLH) transcription factor binding sites. Between 1 and 5 copies of the ABA/WDS and AP2/ERF binding sites are present in each *AtBiP* and *StBiP* promoters (Fig. 5a). Regarding the AP2/ERF transcription factor family binding sites, the BBM, RAP2.1, B3, and ESR1 are differently represented. The B3 CRE was found only in the *AtBiP1* promoter while ESR1 CRE was only present in the *StBiP1* promoter (Supplementary Table S3). Among the bHLH transcription factor binding sites, three CREs are differentially represented. The bHLH28(MYC5) CRE occurs in all Arabidopsis and potato promoters except for *StBiP3*. The bHLH64 is only present in the *AtBiP3* promoter while BEE2 CREs occurs in *StBiP1* and *StBiP3* promoters.

There are 78 bZIP transcription factors in Arabidopsis which generally contribute to seed germination, sugar signaling, amino acid metabolism, salt stress, hypo-osmolarity responses, UPR, and pathogen defense responses. Regarding sucrose regulation and hormone signaling, the bZIP44/53/GBF6, AS1/OCS/TGA-like, and PEND CREs were prominent but differentially represented in the Arabidopsis and potato *BiP* promoters (see Supplementary Table S3 online). Only the *AtBiP3* and *StBiP3* promoters have PEND target CREs^33–35^.

While UPR-related elements were previously identified in the *AtBiP* promoters^36,37^, we compared the UPR element II (UPRE-II), UPRE-III, ERSE-I and ERSE-II in the promoter of *AtBiPs* and *StBiPs* (Fig. 5c, Supplementary Table S3). UPRE-II and ERSE-II elements were overlapping in *StBiP1* and *StBiP2* promoters. The UPRE-III elements were present on *StBiP2* and all *AtBiP* promoters. Only *AtBiP3* and *StBiP3* promoters contain ERSE-I elements that are binding sites for the ER-stress induced AtbZIP28 factor (Fig. 5c)^38,39^.

The WRKY and CAMTA binding sites are well known for pathogen defense, abiotic stress, and the unfolded protein response. The WRKY binding sites were present in *AtBiP1, StBIP1*, and *StBIP2*, but lacking in the *AtBiP3* and *StBiP3* promoters (Fig. 5A and Supplementary Table S3). In particular, WRKY7, -11, and -17 transcription factors are known to control *AtBIP1*/2 expression by suppressing the *AtbZIP28* arm of the UPR during plant immunity^40^. The *StBIP3* has CAMTA binding sites, which are lacking in the *StBiP1/2* promoters.

## Discussion

The molecular chaperone BiP ensures protein homeostasis and is an essential regulator of ER stress transducers in eukaryotes. The characterization of BiPs is crucial to understanding cellular, tissue-specific, and developmentally related responses to ER stress. The *BiP* gene family has been investigated in several plant species, including Arabidopsis, rice, wheat, pepper and citrus^36,37,41–44^. Historic and recent studies using transgenic lines overexpressing various BiPs characterize their roles in plant tolerance to adverse environmental conditions or tissue specific gene expression^2,15,32,36,43,45^. Importantly, we had difficulties retrieving original data relevant to these publications, especially for *Solanaceae* family, because of the lack of proper gene nomenclature, annotation, presence of several genome assemblies, and unavailability of relevant nucleotide sequences in databases (GenBank, ENA, etc.)^10,11,15,36^. To resolve these issues, we retrieved relevant data directly from curated datasets and updated assemblies while cross comparing with the findings of the above-mentioned studies (Table 1 and Supplementary Table S1 online). This study reports a comprehensive phylogenetic analysis of already identified and some novel *BiP*s covering fungi, plant and animal kingdoms in order to identify and characterize BiPs in potato and dissect their evolutionary history.

We report three candidate *BiP*s (*StBiP1*, *StBiP2*, and *StBiP3*) in the *S. tuberosum* genome. Phylogenetic analysis, amino acid sequence analysis, 3-D protein modeling, and gene structure analysis confirmed that these potato gene candidates are true *BiP* genes. The phylogenetic tree divided the *StBiP*s into Group A and Group B, consistent with previous studies (Fig. 1)^37,44,46^. Both groups contained monocot and dicot *BiP*s. Group A contains *AtBiP*3 and *StBiP*3. Group B contains the *StBiP*1 and *StBiP*2 and shows higher diversification compared to Group A. Within Group B, *StBiP*1 and *StBiP*2 diverged more recently from *AtBiP*1 and *AtBiP*2. At the amino acid level, the *StBiP*1 and *StBiP*2, and the *AtBiP*1, *AtBiP*2 were highly similar (92% and 90% respectively) compared to *StBiP*3 and *AtBiP*3 (70–76%). The three StBiP and AtBiP proteins share the expected six protein functional domains that define NBDs and SBDs in eukaryotic BiPs. The 3-D model superimposition also revealed a higher degree of structural similarity between the BiP proteins in Arabidopsis and potato, indicating that their grouping is a result of the variations in non-conserved regions rather than functionally important conserved regions.

Gene structures are reliable indicators of the evolutionary history of gene families^47^. The exon/intron structures and intron numbers vary from zero to eight among the 46 *BiP* genes. Such diversity arises from insertions and deletions during evolution that alters the distance between the start codon and downstream intron peaks. The Group A gene structures and intron phase patterns are conserved except for SORBI_3001G118600. The Group B has 32 members and excluding the *AtBiP1/2*, SORBI_3004G0117700, and the four *BiPs* that lack introns, all other members have similar intron phase patterns throughout the genes. Although the gene structure and phylogenetic studies differentiated *StBIP1, StBiP2*, and *StBiP3* into different groups, functional differences for plant development or the management of ER stress could not be assigned based on gene structure or protein phylogeny. Instead, these data support the functional redundancies often seen in plant experiments where individual *BiP* knockout mutations are partially or fully complemented by other BiP genes^27^.

In Arabidopsis, pepper, and rice, the *BiP* genes were primarily distinguishable by their expression profile^42,43,45^. This study showed that potato BiPs have distinguishable expression patterns. Under normal conditions, *StBiP1* and *StBiP2* are expressed in all tissues, whereas *StBiP3* is only in roots. Treatments with high temperature and salt stress, induce only *StBiP3* expression in leaves. *StBiP1*, *StBiP2*, and *StBiP3* expression was elevated in response to expression of the PVX TGB3 and PVY 6K2 proteins, paralleling results in Arabidopsis for induction of *AtBiP1, AtBiP2,* and *AtBiP3*^48^. These functional studies support the phylogenetic protein diversification of StBiP proteins, despite the highly conserved protein structures.

We propose that the promoter architecture, which controls the gene expression profiles, is key to the BiPs’ contributions to plant development. Understanding the promoter architecture is key to predicting gene functional specificity concerning tissue, timing, and pattern of expression. This study elucidates the various roles of the potato *BiPs* by combining gene expression profiles and analysis of promoter architecture. The frequency and the linear distribution of CREs in the *StBiP* promoter sequences produced two phylogenetic branches that coincide with the separation of Group A (BiP3) and Group B (BiP1/2). The identified CREs correspond to twenty-five transcriptional factor families and suggest fine regulation of BiPs under various developmental and stress-related stimuli. Profiling the distribution and frequency of CREs among *StBiP*s and *AtBiP*s revealed important similarities between the promoter architecture of Group B BiPs.

The *StBiP1* and *StBiP2* promoters are highly enriched with CREs related to cell differentiation, organ development, and hormone-regulated plant development. Considering only the potato BiPs, the presence of MYB3R-like target CREs in the *BiP1/2* genes potentially coordinates the elevated levels of *StBiP1* and *StBiP2* as part of the gene network in shoots, stems, stolons, tubers, and stamens^64,65^. The *AtBiP2* is the only promoter where we identified WEREWOLF CREs (MYB target) that play a major role in epidermal cell patterning and WRKY7/57 CREs that contribute to stress responses (Supplementary Table S3 online)^49–51^. The profile of CREs in *AtBiP1* and *AtBiP2* identified differences that could not be otherwise detected in gene expression assays using gene specific probes, because of their extensive sequence identity. These data suggest that *AtBiP1* and *AtBiP2* have overlapping and unique functions.

The *StBiP3* promoter has fewer CREs involved in cell differentiation and organ development and is enriched with stress related CREs (Fig. 5a, c and Supplementary Table S3 online). The varied distribution of the root specific ROOTMOTIFTAPOX1 motif among all the *StBiP* promoters, likely contributes to different gene expression levels in root tissues. All *StBiP* promoters contain CREs representing the AP2, bHLH, ABA/EDS transcription factor families that link development, hormonal regulation, and stress response in plants. Some of the identified CREs recognized by AP2 transcription factors include B3 (mediation of auxin and brassinosteriod dependent development transitions), BBM and RAP2.6 (in cell proliferation, cell morphogenesis, pathogen infection, salt stress, and osmotic stress responses), and RAP2.1 (dehydration response)^38–40,45,47,48,52^.

All three *StBiP* promoters contain various bZIP transcription factor binding CREs important for biotic and abiotic stress-mediated signaling in plants. The presence of the AS1/OCS/TGA type elements, which are components of regulatory modules associated with hormonal regulation (auxin, salicylic acid, and jasmonic acid) under biotic stresses, wounding, and oxidative stress, might partially explain the activation of all BiP genes by biotic stressors. This study shows that *StBIP1, StBiP2* and *StBIP3* are consistently induced in response to PVY 6K2 which is an known activator of ER stress related gene expression^48,53^ The linear pattern of ER-stress related UPRE and ERSE elements in *AtBiP* and *StBiP* promoters potentially contribute to the differential expression of BiPs under normal and stressed conditions, and could explain the stress-responsive role of AtBiP3 and StBiP3.

Collectively, the promoter analysis results provide the ability to predict when, where, how, and how much each *BiP* can be expressed in potato. Overall, the identification of *cis*-regulatory architecture will extend our understanding of the complex developmental and stress related *cis*–*trans* relationships involved in the regulation of *BiP* genes. This analysis provides a foundation for future studies to dissect the components of transcriptional regulation of *BiP* genes using in vitro and in vivo strategies including Electrophoretic Mobility Shift Assay (EMSA), Chromatin Immunoprecipitation Sequencing (ChIP-Seq), promoter deletion, and other transgenic approaches for identifying condition-specific combinations of CRE modules. We anticipate further investigations will be useful for the functional characterization of *BiP*s, and for developing transgene-free crops that can withstand stress conditions using both classical and modern genome editing strategies.

In summary, we identified three BiPs in potato and named them based on their sequence similarity to Arabidopsis BiPs. Their gene structure and protein domain architectures were highly conserved during evolution, leading us to further investigate the basis for their distinguishable and sometimes overlapping roles in plant growth, development, and stress response. Figure 5a reveals that the differences between *BiP* promoter sequences produced phylogenetic branches that cluster *StBiP1*, *StBiP2,* and *AtBiP2* as well as *StBiP3* and *AtBiP3* into separate groups. The pattern of CREs appears to be responsible for promoter differences which correlate with the different gene expression patterns observed between *BiP1/2* and *BiP3*. Based on our findings, we propose a model in which the promoter architecture drives the spatiotemporal regulation of BiPs for plant growth, development, and stress responses. Accordingly, the distribution and frequency of various developmental, stress-responsive and tissue-specific CREs can be considered as the primary determinant of the fine regulation of BiPs both in Arabidopsis and potato.

## Methods

### Genome-wide identification of the *BiP*s in potato and phylogenetic analysis

We used BLASTP and the annotated Arabidopsis *BiP* genes (*AtBiP1*-*3*; AT5G28540.1, AT5G42020.2 and AT1G09080.2) which we retrieved from The Arabidopsis Information Resource 10 (TAIR10) (https://www.arabidopsis.org) to search for BiP homologs in the assemblies of *Brachypodium distachyon* (v3.0), *Capsicum annuum* (ASM51225v2), *Glycine max* (v2.1) *Nicotiana attenuate* (NIATTr2), *Nicotiana benthamiana* (v1.0.1), *Nicotiana tabacum* (v4.5), *Oryza sativa* (IRGSP-1.0), *Sorghum bicolor* (NCBIv3), *Solanum lycopersicum* (SL3.0), *Solanum tuberosum* (in the SolTub_3.0 Genome Assembly), *Triticum aestivum* (IWGSC), *Zea mays* (B73_RefGen_v4), assembly available at the Sol Genomics Network database and the Ensembl Plants database (release 43)^17–19^ within the publicly available Ensembl Genomes project^54^. An e-value threshold of 10^–25^ was used for the development of the first list of BiPs which included already annotated BiPs as well as candidate sequences of species in *Solanaceae*. Then subcellular localization of the protein sequences of identified potential candidates was assessed using DeepLoc-1.0^55^. Only proteins with ER localization signals were identified as putative BiPs. The ENSEMBL Locus IDs were then used to retrieve the gene names and other identifiers from NCBI, UniPro, and TAIR databases (see Supplementary Table S1 online). Multiple sequence analysis was carried out using MUSCLE built into Seaview (v 4.7). The phylogenetic tree was generated using default settings of PhyML (v 1.5) build into Seaview (v 4.7) (https://doua.prabi.fr/software/seaview) with 1,000 bootstrap iterations^56^. The phylogenetic tree was visualized using iTOL (v4)^57^. Then Adobe Photoshop CC (2017) to compile diagrams into figures and provide labels and add color coding.

### DNA extraction and sequencing

Rooted cuttings were grown in soil inside chambers with a 16 h photoperiod at 22 °C for 3 weeks. Leaf tissues were harvested in liquid nitrogen and ground using a motor and pestle. DNA extraction and purification were carried out using the Mini Genomic DNA Kit (IBI Scientific, Peosta, Iowa, USA). DNA was quantified using the Epoch spectrophotometer (Biotek, Winooski, VT) Powerwave XS2 with Gen 5.0 software. StBiP1, StBiP2, and StBiP3 genomic sequences were PCR amplified using gene-specific primers (Supplementary Table S4 online). Platinum SuperFi Green DNA Polymerase (Invitrogen) mix was used with PCR amplification conditions as follows; Initial denaturation 98 °C for 2 min, 98 °C 10 s, 60.9 °C 10 s, 72 °C for 2 min for 35 cycles, and final extension of 72 °C for 5 min. Amplified products were visualized using agarose gel (1%) electrophoresis. Sequencing was carried out using ABI 3,130 Genetic Analyzer (Applied Biosystems) using sequencing primers (Supplementary Table S4 online). The sequencing data was analyzed using Geneious Prime v. 2019.2.1. Gene sequences were deposited at NCBI Genbank under following accession numbers; MN982518 (*StBiP1*), MN982519 (*StBiP2*) and MN982520 (*StBiP3*).

### Gene structures, domain analyses, and protein structure analyses

Intron–exon structures and intron phases of BiPs were visualized using GSDS 2.0 (https://gsds.cbi.pku.edu.cn/)^58^. Conserved domain analysis and visualization were carried out using Geneious Prime (v. 2019.2.1). Images were downloaded from GSDS 2.0. The I-Tasser structure and function prediction tool (Zhang lab) was used for structural modeling of the protein sequences. PyMol (v1.7.4) (https://pymol.org/2/) was used to visualize the protein domains of the chosen models and for the superimposition of the models. Selected mages were downloaded and then constructed into figures using Adobe Photoshop CC (2017).

### Gene expression analysis based on the RNA-seq data

Transcriptomic analysis was carried out using RNA-seq data (accession number E-MTAB-552) generated by Potato Genome Sequencing Consortium (PGSC) available at the Expression Atlas Database (https://www.ebi.ac.uk/gxa/home)^24^. Transcripts per million (TPM) were calculated using raw counts by averaging technical replicates followed by quantile normalization of biological replicates using Limma^59^. TPMs were used to generate heatmap using MultiExpression Viewer (MeV) version 4.9.0^60^. The diagrams and charts were compiled in Adobe Photoshop CC (2017).

### Plant materials and stress induction

*Solanum tuberosum* cultivar ‘Russet Norkota’ were vegetatively multiplied in vitro on Murashige and Skoog (MS) medium (PhytoTech Labs, Lenexa, KS) or by cuttings placed into soil. All plants were grown inside chambers with a 16 h photoperiod at 22 °C for three weeks. The in vitro propagated plants were used for temperature stress experiments. These plants were subjected to an overnight 16 h at high (37 °C) or low (4 °C) temperature. Since the incubators for high and cold temperatures did not have lights, we subjected the control plants grown at regular (22 °C) temperature to an overnight 16 h of dark conditions to maintain consistent light conditions among the control and experimental plants. Leaves were harvested after the 16 h treatment for RNA extraction (below). Experiments requiring infiltrating leaves with solutions of DTT, NaCl or agrobacterium were carried out using rooted cuttings in soil according to Henriquez-Valencia et al.^30^. For abiotic stress, 2 mM dithiothreitol (DTT), 150 mM sodium chloride (NaCl) or H_2_O as mock treatment were infiltrated into leaves with a 1 mL needle-free syringe. Then leaf samples were harvest at 4 h post infiltration for RNA extraction and RT-qPCR (see below). For viral protein stress, PVX TGB3 and PVY 6K2 sequences were cloned by Gateway Technology (ThermoFisher) in pGWB505 binary vector for produce C-terminal GFP fusions. All plasmids were sequence verified and maintained in *Agrobacterium tumefaciens* sp. strain GV3101. *A. tumefaciens* carrying the GFP expression vector pXF7FNF2.0 was used as a mock control. *A. tumefaciens* harboring TGB3-GFP, 6K2-GFP or GFP were collected and suspended in a solution of 10 mM MES-KOH (pH 5.6), 10 mM MgCl_2_, 200 μM acetosyringone and adjusted to OD_600_ = 0.7. Then potato leaves (three weeks after rooting) were infiltrated with 1 mL needle-free syringe. These treated leaves were harvest at 4 days post infiltration for RNA extraction (below).

### RNA extraction and RT-qPCR

Leaves were harvested, ground in liquid nitrogen, and RNA was extracted using the RNeasy Plant Mini Kit (Qiagen, Germantown, MD). Total RNA (2 µg) was reverse-transcribed using a High-capacity cDNA Reverse Transcription Kit (Applied Biosystems, Foster City, CA) and random primers were employed for cDNA synthesis. The cDNAs were diluted 500 × and then qPCRs were performed using the SYBR Green Master Mix (Applied Biosystems). The qPCR amplification was performed for *StBiP1*, *StBiP2*, and *StBiP3* using gene specific primers (Supplementary Table S5 online). The qPCR amplification of *StACTIN58* (PGSC0003DMG400023429) was carried out as an internal control using gene specific primers (Supplementary Table S5 online). The ΔΔCt method was used for calculating relative gene expression. The data represent the mean of two technical replicates for three biological repetitions. Data was analyzed and charted using Microsoft Excel (2019). Charts were compiled using Adobe Photoshop CC (2017).

### Confocal microscopy to confirm expression of viral proteins fused to GFP.

Leaf segments were placed on microscope slides and protected by cover slips before examination with an Olympus Fluoview FV1000 confocal laser scanning microscope and a 60 × objective (Olympus America Inc. Center Valley, PA). Laser excitation wavelength of 515 nm and Z-dimensions of 5 µm with 0.5 µm/slice. Image J 1.52p software^61^ was used for stacking Z-series images.

### Ab initio promoter analysis

Promoter sequences representing 2000 bp from the transcription start site were retrieved from the Ensemble Plants database (https://plants.ensembl.org/; Ensembl Plants release 43, April 2019). CREs were identified using the New PLACE database (https://www.dna.affrc.go.jp/PLACE/), the TRANSFAC database (release 2019.2 https://genexplain.com/transfac/) and the Catalog of Inferred Sequence Binding Proteins (CIS-BP) Database (v 2.00) (https://cisbp.ccbr.utoronto.ca/) on 1,000 bp from the transcription start site^62–64^. Hierarchical clustering was carried out using the Manhattan correlation with the average linkage method in MultiExpression Viewer (MeV) (v4.9.0) https://mev.tm4.org/)^60^. The output of the hierarchical clustering was retrieved and the heatmap was recreated using Microsoft Excel (2019). UPRE and ERSE elements were identified in the 2000 bp promoter sequence (see Supplementary Table S3 online). Development and tissue-specific expression profiles were obtained from the Expression Atlas Database (https://www.ebi.ac.uk/gxa/home)^24^. Geneious Prime (v. 2019.2.1) was used to annotate and visualize the CREs and the diagrams were compiled using Adobe Photoshop CC (2017).

## Supplementary information

Supplementary file1 (PDF 1880 kb)
