# Supplementary material for: Genome-wide identification and characterization of Solanum tuberosum BiP genes reveal the role of the promoter architecture in BiP gene diversity
Source: Sci Rep. 2020 Jul 9;10:11327. doi: 10.1038/s41598-020-68407-2 (PMC7347581; doi:10.1038/s41598-020-68407-2)
Supplement: Supplementary file 1 — Supplementary file1 (PDF 1880 kb) [file 41598_2020_68407_MOESM1_ESM.pdf]

Supplementary Information

Genome-wide identification and characterization of *Solanum tuberosum BiP* genes reveals the role of the promoter architecture in BiP gene diversity.

Venura Herath[abd], Mathieu Gayral[a], Nirakar Adhikari[c], Rita Miller[c], Jeanmarie Verchot[ab#]

[a] Texas A&M Agrilife Center in Dallas, Dallas, TX 77953
[b]Department of Plant Pathology & Microbiology, Institute for Plant Genomics and Biotechnology, Texas A&M University, College Station, TX 77802
[c] Department of Biochemistry and Molecular Biology, Oklahoma State University, Stillwater, OK 77845
[d] Department of Agriculture Biology, Faculty of Agriculture, University of Peradeniya, Sri Lanka

#Corresponding author: Department of Plant Pathology & Microbiology, Institute for Plant Genomics and Biotechnology, Texas A&M University, College Station, TX 77802. Tel: 979-845-1788. Email: jm.verchot@tamu.edu

Table S1. Molecular characterization of additional genes used for phylogeny[a]

Kingdom	Clade	Family	Species	Locus ID	Gene Name[b]	Transcript ID	Protein Id[c]	MW (kDa)	PI	Length (bp)
Plantae	Eudicots	Fabaceae	*Glycine max*	GLYMA_05G219400	BiP4, soyBiPD	KRH60086	NP_001238736.2	63.67	6.57	581
				GLYMA_05G219600	BiP3	KRH60091	NP_001344270.1	77.64	5.22	702
				GLYMA_08G025700	BiP5	KRH41370	XM_003530405.3	73.48	4.78	668
				GLYMA_08G025900	BiP2	KRH41372	NM_001248012.2	78.06	4.84	709
		Poaceae	*Brachypodium distachyon*	BRADI_3g01477v3	BiP3	KQJ92901	KQJ92901	73.36	4.79	665
			Oryza sativa	Os02g0115900	BiP1	Os02t0115900-01	Q6Z7B0.1	73.4	4.79	665
				Os03g0710500	BiP2	Os03t0710500-00	Q53RJ5.1	73.38	5.11	669
	Monocots			Os05g0367800	BiP3	Os05t0367800-00	Q6L590.1	72.22	5.01	669
				Os05g0428600	BiP4	Os05t0428600-01	Q75HQ0.1	74.31	4.8	687
				Os08g0197700	BiP5	Os08t0197700-00	Q6Z058	83.24	5.13	764
			Sorghum bicolor	SORBI_3004G011700	hypothetical protein	KXG29293	KXG29293	71.49	4.91	649
				SORBI_3003G378700	hypothetical protein	EES01866	EES01866	73.3	4.71	667
				SORBI_3001G118600	hypothetical protein	EER93587	EER93587	74.48	5.13	676
			Triticum aestivum	KC894715	TaBiP1	KC894715.1	AGN94840.1	73.19	4.81	665
				KC894716	TaBiP2	KC894716.1	AGN94841.1	73.22	4.81	665
				KC894717	TaBiP3	KC894717.1	AGN94842.1	73.17	4.81	665
			Zea mays	Zm00001d014993	hypothetical BiP	Zm00001d014993_T001	P24067.3	73.085	4.8	663
				Zm00001d054043	hypothetical BiP	Zm00001d054043_T003	AQK61113.1	73.13	4.83	663
Animalia		Hominidae	*Homo sapiens*	ENSG00000044574	HSPA5-201 Hs, GRP78	ENST00000324460.7	P11021	72.33	4.77	654
		Muridae	*Mus musculus*	ENSMUSG00000026864	HSPA5-201 Mn, GRP78	ENSMUST00000028222.12	P20029	72.42	4.77	655
			Rattus norvegicus	ENSRNOG00000018294	HSPA5-201 Rn, GRP78	ENSRNOT00000025067.6	P06761	72.35	4.77	654
Fungi		Saccharomycetaceae	*Saccharomyces cerevisiae*	YJL034W	KAR2	YJL034W_mRNA	CAA89325.1	74.47	4.48	682

[a] Locus ID, Transcript ID, MW, PI and Length were obtained from ENSEMBL

[b]Gene names were obtained from ENSEMBL, NCBI or UniPro databases.

[c]Protein IDs are compiled from TAIR for Arabidopsis, NCBI and UniPro for all other sequences

Table S2: I-TASSER modeling results of yeast, Arabidopsis and potato BiPs

Protein	C-score[a]	Exp. TM score[b]	Exp. RMSD[b]	No. of decoys	Cluster Density[d]	Templates with high Z-score (PDB ID)[d]
Kar2 (YJL034W)	0.01	0.71+ -0.11	8.0+ -4.4	2043	0.1349	5e84A (P11021)
AT1G09080	0.26	0.75+ -0.10	7.4+ -4.2	2079	0.1715	5e84A (P11021)
AT5G28540	0.09	0.73+ -0.11	7.8+ -4.4	2063	0.1442	5e84A (P11021)
AT5G42020	-0.16	0.69+ -0.12	8.1+ -4.4	1723	0.1107	6eofA (G3I8R9)
PGSC0003DMG400024707	0.46	0.77+ -0.10	6.9+ -4.1	2097	0.2087	5e84A (P11021)
PGSC0003DMT400031937	0.07	0.72+ -0.11	7.8+ -4.4	2077	0.1422	5e84A (P11021)
PGSC0003DMT400047710	0.07	0.72+ -0.11	7.8+ -4.4	2050	0.1424	5e84A (P11021)
PGSC0003DMT400050462	0.65	0.80+ -0.09	5.9+ -3.7	2099	0.3166	5e84A (P11021)

[a] The quality of the predicted model is estimated by the Confidence (C) -score which has a value ranging between -5 and 2. A C-score >-1.5 indicates low error quality between the estimated and observed structure models.

[b] TM(Template-modeling)-score and RMSD (Root mean square deviation) are the measures of accuracy of the structure modeling when compared with native structure. The TM-score measures the topological similarity between protein and template models. Correct topology of the model is represented when the TM score is bigger than 0.5. A TM-score lower than 0.17 represents random similarity between predicted model and native structure. All the models presented here have TM value higher than 0.5. The average distance between all residue pairs in two similar structure is represented as RMSD. TM and RMSD values represented here are based on their correlation with C-score. The TM score measures the quality of the query model and the native structure of the template. Each tested protein has a TM score >0.5 indicating the topology of the model is correct.

[c] The Cluster density signifies the model quality. A Higher cluster density value means a better model quality. Technically, the cluster density is the density of decoys per unit space. Decoys are the replicas of peptide chain fragments generated during simulation to generate full atomic models of the protein.

[d] The templates here are the Human BiP (P11021) and Chinese Hamster (G3I8R9)

Table S3. Key CREs in plant BiP promoters that engage in cytoprotective responses to environmental challenges

Group	Gene	ABA/WDS	AP2	ARF	ARR-B	BBR/BPC	BES-1	bHLH	bZIP	C2H2	CAMTA	CCT	DOF-ZnF	E2F/DP	EIL	FAR1	GATA	HB/HD-ZIP	HMG	HSF	LOB	MADS	MYB	NAC	SBP	SHI	SPL	SRS	TCP	Trihelix	WRKY
A	Os03g0710500	5	5	2	0	1	0	6	4	0	2	1	2	0	0	1	1	0	0	2	6	1	9	6	0	0	4	0	2	1	1
	SORBI_3001G118600	5	5	0	1	0	0	3	4	1	1	3	0	1	0	2	0	0	1	9	2	0	3	6	0	0	3	0	0	1	0
	T459_34102	2	1	1	0	0	0	1	3	1	2	1	10	0	0	0	1	0	0	2	0	0	5	3	0	0	1	0	1	2	1
	Solyc01g099660.3	1	3	2	0	0	0	0	3	0	1	1	0	0	0	1	1	0	1	4	0	2	3	2	0	0	0	0	0	2	1
	A4A49_38137	1	0	1	6	0	0	0	2	0	1	2	1	0	0	0	0	0	1	0	6	1	8	0	0	0	3	0	0	0	1
	Nitab4.5_0004891g0060.1	5	2	0	1	0	0	2	3	0	0	0	1	1	0	0	0	0	0	2	1	2	3	1	0	0	0	0	0	0	0
	Nitab4.5_0003658g0020.1	0	2	0	0	0	0	3	2	1	0	1	1	0	0	0	1	0	0	4	2	2	7	3	0	0	2	0	0	0	2
	Niben101Scf00369g07016.1	1	2	6	1	0	0	2	2	1	0	4	8	1	0	0	2	3	0	2	0	1	7	0	0	0	0	0	0	1	1
B	SORBI_3003G378700	2	7	0	0	0	0	3	5	0	1	1	0	0	0	1	0	0	2	7	4	0	3	7	0	0	3	0	0	1	3
	Os05g0428600	0	9	0	0	0	0	3	4	0	1	3	1	0	0	1	1	0	0	2	6	2	7	7	0	0	3	0	0	0	1
	Os05g0367800	2	21	0	1	0	0	4	7	0	6	3	3	0	1	6	2	1	0	3	13	0	5	7	0	0	2	0	3	0	1
	Os08g0197700	1	2	0	1	0	0	4	1	0	0	4	0	0	0	0	1	1	0	0	1	1	6	3	0	0	4	0	0	1	3
	Os02g0115900	2	9	2	0	0	0	2	8	0	2	2	1	0	0	0	1	4	0	3	4	1	9	3	0	0	0	0	1	1	0
	SORBI_3004G011700	0	11	2	0	0	0	1	3	1	7	1	1	0	1	3	2	2	0	3	8	0	1	10	0	0	1	0	2	0	0
	Zm00001d014993	0	9	0	1	0	0	2	4	0	5	2	0	0	0	2	1	1	0	7	11	1	3	3	0	0	1	0	1	1	0
	Zm00001d054043	2	9	2	1	0	0	3	4	0	4	1	2	0	0	2	1	1	0	2	9	2	5	6	0	0	1	0	2	0	0
	BRADI_3g01477v3	2	8	1	1	0	0	2	8	0	1	1	1	0	0	1	0	1	0	2	6	0	6	6	0	0	3	0	0	0	0
	KC894716	1	11	2	2	0	0	5	9	1	4	1	0	0	0	5	0	0	3	6	16	1	3	6	0	0	5	0	4	1	1
	KC894715	0	14	4	1	0	0	4	7	0	7	1	1	0	0	8	0	0	1	4	13	1	5	7	0	0	3	0	6	0	0
	KC894717	3	16	4	0	0	0	4	3	1	5	2	1	0	0	3	2	0	2	5	19	0	7	4	0	0	2	0	3	0	0
	GLYMA_08G025700	0	3	0	0	0	0	0	1	0	0	3	2	0	0	0	1	3	0	3	0	0	1	1	0	0	0	0	1	0	0
	GLYMA_05G219400	1	3	3	0	0	0	0	2	0	1	2	2	1	0	0	2	2	0	2	0	0	3	2	0	0	0	0	1	0	1
	GLYMA_05G219600	1	1	0	1	1	0	0	1	0	0	2	4	0	0	0	0	1	0	0	0	0	5	0	0	0	0	0	0	0	0
	GLYMA_08G025900	1	1	1	0	0	0	2	1	0	0	4	1	0	0	0	0	2	1	1	0	1	3	0	0	0	0	0	0	1	0
	T459_00063	0	3	0	1	0	0	3	4	0	0	1	0	0	0	0	2	1	0	3	1	2	1	4	0	0	2	0	0	1	2
	Solyc08g082820.3	0	1	1	1	0	0	2	3	0	1	1	1	0	0	1	1	0	0	0	3	0	3	3	0	0	3	0	0	1	0
	A4A49_23212	0	3	1	2	0	0	1	6	0	1	3	0	0	0	0	1	1	0	1	3	1	7	3	0	0	2	1	1	0	1
	A4A49_57922	2	1	1	0	0	0	3	6	0	4	2	2	0	0	1	3	0	0	2	0	2	5	1	1	0	3	0	0	0	1
	Niben101Scf02972g05008.1	1	2	2	0	0	1	2	6	0	1	1	2	0	0	0	0	1	0	1	0	1	1	0	0	0	0	0	2	0	1
	Nitab4.5_0009965g0010.1	1	5	1	0	0	0	2	6	0	4	4	0	1	0	1	6	0	0	2	1	2	6	5	0	0	3	0	0	2	0
	Niben101Scf02755g06016.1	0	6	1	0	0	0	2	5	0	0	4	1	1	0	0	2	1	0	0	1	1	5	3	0	0	2	0	2	0	0
	Niben101Scf03115g02008.1	0	12	1	0	0	0	2	6	1	1	2	0	1	0	1	1	1	0	5	1	2	9	5	0	0	3	0	0	2	1
	A4A49_65725	0	1	0	2	0	0	1	6	0	1	1	1	0	0	1	2	0	0	3	2	0	9	1	0	0	2	1	0	0	0
	Nitab4.5_0005771g0020.1	2	1	0	0	0	0	1	9	1	0	3	0	0	0	1	0	2	3	4	0	9	0	2	0	0	1	0	0	1	0
	T459_09640	1	1	1	0	0	0	0	5	0	0	3	5	0	0	0	4	1	0	4	2	0	2	2	0	0	1	0	1	1	0
	Solyc03g082920.3	2	4	1	0	0	0	1	7	0	0	1	0	1	0	0	2	2	1	1	1	0	2	0	0	0	1	1	1	1	0

Table S4: Key CREs in StBiP and AtBiP promoters that engage in cytoprotective responses to environmental challenges

Transcription Factor Family[a]	CRE	Consensus Sequence	StBiP1	StBiP2	StBiP3	ATBiP1	ATBiP2	ATBiP3
MYB	MYB3R-like	NWWWNTNACCGTTRN, NWWWNTRACCGTTRN, NAAWWNTNACCGTTR5	X	X	X	X	X	-
	MYB24	NNGTTAGGY[6–8]	X	-	X	X	X	-
	WEREWOLF	AACTAACAG[9–13]	-	-	-	-	X	-
AP2/ERF	BBM	GGGCGCCANT[14,15]	-	X	X	-	-	X
	RAP2.1	NTGTCGGTGGNGN[5]	-	X	-	-	X	X
	B3	CTGCATGCN[6]	-	-	-	X	-	-
	ESR1	NGGCCGCCCG[14,15]	X	-	-	-	-	-
bHLH	bHLH28 (MYC5)	NNNTGTACGGAN[5]	X	X	-	X	X	X
	bHLH64	ACCAGT[5]	-	-	-	-	-	X
	BEE2	NNCACGTGNN[6,7,16]	X	-	X	-	-	-
bZIP	bZIP44/53/GBF6	NNWGCTGACGTGGCA[5], NNTGCTGACGTGGCA[5], TGCCACGTCAGCANN[5]	X	X	-	X	-	X
	AS1/OCS/TGA-like	AATTTGAT, TAATTTGA[1,2]	X	X	X	-	X	X
	PEND	ANTTCTTATK[3,4]	X	-	X	-	-	X
unfolded protein response bZIP factors	ERSE I	CCAATNNNNNNNNNCACG[1]	-	-	X	-	-	X
	ERSE II	ATTGGNNCACG[1]	X	X	-	-	-	-
	UPRE I	TGACGTGR[1]	-	-	-	-	-	-
	UPRE II	GATGACGCGTAC[2]	X	X	-	-	X	-
	UPRE III	TCATCG[3]	-	X	X	X	-	X
WRKY	WRKY7/57	NNCGTTGACTTTTN[5], NNRGTCAAMN[6,7,17]	X	X	-	-	X	-

[a] All CREs and associated transcription factor famileswere identified using the NEW PLACE, TRANSFAC, and CIS-BP build databases except for the CREs involved in the unfolded protein response These elements are recognized by bZIP factors and were manually identified in the promoters.

References:

1. Yun, K.-Y. *et al.* Transcriptional regulatory network triggered by oxidative signals configures the early response mechanisms of japonica rice to chilling stress. *BMC Plant Biol.* **10**, 16 (2010).

2. Garretón, V., Carpinelli, J., Jordana, X. & Holuigue, L. The as-1 promoter element is an oxidative stress-responsive element and salicylic acid activates it via oxidative species. *Plant Physiol.* **130**, 1516–26 (2002).

3. Portales-Casamar, E. *et al.* JASPAR 2010: The greatly expanded open-access database of transcription factor binding profiles. *Nucleic Acids Res.* **38**, 105–110 (2009).

4. Mertin, S., McDowall, S. G. & Harley, V. R. The DNA-binding specificity of SOX9 and other SOX proteins. *Nucleic Acids Res.* **27**, 1359–64 (1999).

5. O'Malley, R. C. *et al.* Cistrome and Epicistrome Features Shape the Regulatory DNA Landscape. *Cell* **165**, 1280–1292 (2016).

6. Mathelier, A. *et al.* JASPAR 2016: A major expansion and update of the open-access database of transcription factor binding profiles. *Nucleic Acids Res.* **44**, D110–D115 (2016).

7. Weirauch, M. T. *et al.* Determination and inference of eukaryotic transcription factor sequence specificity. *Cell* **158**, 1431–1443 (2014).

8. Zhong, R. & Ye, Z. H. MYB46 and MYB83 bind to the SMRE sites and directly activate a suite of transcription factors and secondary wall biosynthetic genes. *Plant Cell Physiol.* **53**, 368–380 (2012).

9. Kang, Y. H. *et al.* The MYB23 gene provides a positive feedback loop for cell fate specification in the arabidopsis root epidermis. *Plant Cell* **21**, 1080–1094 (2009).

10. Ishida, T. *et al.* Arabidopsis TRANSPARENT TESTA GLABRA2 is directly regulated by R2R3 MYB transcription factors and is involved in regulation of GLABRA2 transcription in epidermal differentiation. Plant Cell 19, 2531–2543 (2007).

11. Tominaga, R., Iwata, M., Okada, K. & Wada, T. Functional analysis of the epidermal-specific MYB genes CAPRICE and WEREWOLF in Arabidopsis. *Plant Cell* **19**, 2264–2277 (2007).

12. Koshino-Kimura, Y. *et al.* Regulation of CAPRICE transcription by MYB proteins for root epidermis differentiation in Arabidopsis. *Plant Cell Physiol.* **46**, 817–826 (2005).

13. Wang, S. *et al.* Control of Plant Trichome Development by a Cotton Fiber MYB Gene. *Plant Cell* **16**, 2323–2334 (2004).

14. Gabdoulline, R., Eckweiler, D., Kel, A. & Stegmaier, P. 3DTF: A web server for predicting transcription factor PWMs using 3D structure-based energy calculations. *Nucleic Acids Res.* **40**, 180–185 (2012).

15. Alamanova, D., Stegmaier, P. & Kel, A. Creating PWMs of transcription factors using 3D structure-based computation of protein-DNA free binding energies. *BMC Bioinformatics* **11**, (2010).

16. Toledo-Ortiz, G., Huq, E. & Quail, P. H. The Arabidopsis Basic/Helix-Loop-Helix Transcription Factor Family. *Plant Cell* **15**, 1749–1770 (2003).

17. Yu, D., Chen, C. & Chen, Z. Evidence for an important role of WRKY DNA binding proteins in the regulation of NPR1 gene expression. *Plant Cell* **13**, 1527–40 (2001).

Table S5: Primer used for the amplification and sequencing

**Sequencing Primers**	
**Primer Name**	**Forward Primer**
StBiP1-S-1	GCATTGGAATAGAATAGTCGATGG
StBiP1-S-2	CTACCGAATAGCCTGTATATTGAC
StBiP1-S-3	TGATTGTGTATTTGTCGTGC
StBiP1-S-4	ATTGACAATGGCGTGTTTGAG
StBiP1-S-5	TTTCTCTGAACCATTGACCC
StBiP1-S-6	AAGTCCCAGGTCTTCACCAC
StBiP2-S-1	AGACCTCTGATACACAGCCG
StBiP2-S-2	CATAACTGGTAGAGGATAAGTACC
StBiP2-S-3	GGGTTGACACCTTTGCTAGG
StBiP2-S-4	AGATGAGTGTCTCCATTTGTG
StBiP2-S-5	ATAGGGAACAAGTTTCATGTCC
StBiP2-S-6	AATCTTCCTAGACATTGTTGGG
StBiP3-S-1	CCGTTCCATGAGGGTGTTAGAG
StBiP3-S-2	AGAGATTAATTGGAGAAGCTGC
StBiP3-S-3	TTAATGATGCACAAAGGCAAGC
StBiP3-S-4	AAGAAGGCATTAGAGGATGC
StBiP3-S-5	GTTTGACATCAGATTGAAGTCTGG
StBiP3-S-6	CATGAAGAACACAATCAACGAC
**Amplification Primers**	
StBiP1-G-F	GGGTCTTTCTTCAGTTTCTCACAGT
StBiP1-G-R	ATTAAAAGGAAATCCTAAATTTTGATGG
StBiP2-G-F	GAGTGACATTGTTCGTCAAAATTC
StBiP2-G-R	AGTTGAAGTTGGTTGCCTTAAAAA
StBiP3-G-F	GGTAATTGGCTGATTGATGTGTAA
StBiP3-G-R	TCACACAAAAATACAGTCCTTGGT
**Sequencing Primers**	
StBiP1-F	GAGGGTGGAGACGAAACTAAAG
StBiP1-R	GGGACTTCTTGGTTGGGATAA
StBiP2-F	GAGGGTGGAGACGAAACTAAAG
StBiP2-R	GCCTTCTTGACAGGAGTCATT
StBiP3-F	CTAGTGACAAGTCGCGTCTAA
StBiP3-R	GAGCATCCACCTTCTCCTTTAC
StACTIN58-F	ACCATCGGTGCTGAACGATT
StACTIN58-R	TCTATCGGCAATACCGGGGA

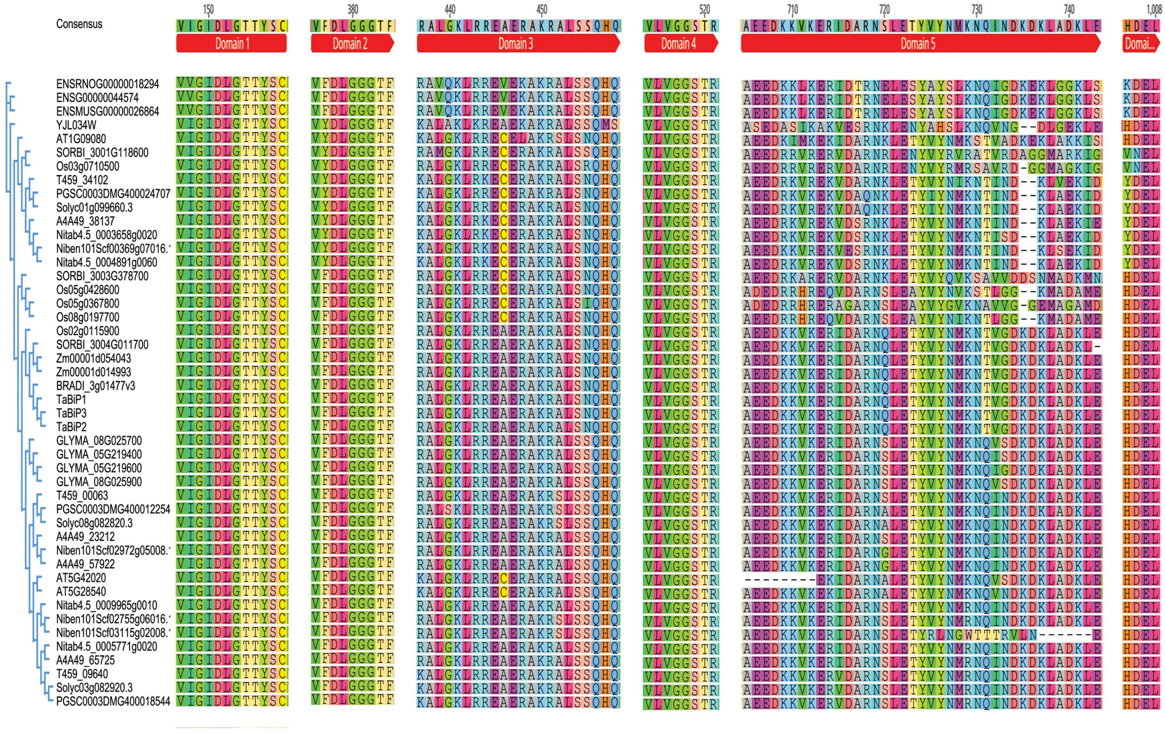

Figure S1: Amino acid sequence alignment generated using MUSCLE built into Seaview (v 4.7). The six conserved amino acid domains in StBiPs shown by amino acid sequence alignment with orthologs in yeast, plant and animal kingdoms. Domain1- β motif; Domain2-γ motif; Domain3 - calmodulin-binding site; Domain4- adenosine-binding motif; Domain5 - αβ motif; Domain6 - ER retention signal – H/K/VDEL.
